# Pattern of Change of Depressive Disorder over a One-Year Period among Community-Dwelling Older Adults in Québec

**DOI:** 10.1155/2013/451708

**Published:** 2013-03-31

**Authors:** Djemaa-Samia Mechakra-Tahiri, Micheline Dubé, Maria Victoria Zunzunegui, Michel Préville, Djamal Berbiche, Joëlle Brassard

**Affiliations:** ^1^Centre de Recherche Hôpital Charles LeMoyne, Campus de Longueuil, Montréal, QC, Canada J4K 0A8; ^2^Laboratory of Gerontology, Department of Psychology, Université du Québec à Trois-Rivières, QC, Canada G9A 5H7; ^3^Faculty of Medicine, Department of Social and Préventive Medicine, Université de Montréal, QC, Canada H3C 3J7; ^4^Faculty of Medicine, Department of Community Health, Université de Sherbrooke, QC, Canada J1H 5N4

## Abstract

The objective of this study was to describe changes in depression and its correlates, in community-dwelling elderly, over a 12-month period. Data come from a longitudinal ESA Study (Enquête sur la Santé des Aînés) of elderly persons (*n* = 2752). Depression was measured using the DSM-IV criteria. Polytomous logistic regression was used to assess relations, over time, between participant's characteristics and depression. Among the 164 (5.9%) subjects, who were depressed at baseline, 19.5% were continuously ill cases and 80.4% had recovered, 12 months later. In polytomous regression, factors increasing the probability of the three depression states (persistence, recovery, and incidence) were daily hassles, stress intensity, and fair/poor self-rated mental health. Depression in old age is dynamic. Available prognostic factors can be taken into account to help direct treatment to elderly at highest risk of a poor prognosis.

## 1. Introduction 

Depression in later life is a major health problem because of its enormous impact on well-being and daily functioning. Depression has been shown to be associated with functional impairment, morbidity, and mortality [1–4] and with greater utilization of health services [4, 5]. Several studies have followed the trajectory of individuals diagnosed with depression, but in a clinical setting [6, 7], and the results show that recovery from depression varies widely between studies. 

A meta-analysis on the prognosis of depression at 24 months in elderly community and primary care populations estimated that only 33% of subjects were well, 33% were depressed, and 21% had died [8]. In a one-year followup, a Canadian study of 380 elderly medical inpatients with major, minor, or no depression [9] found that 13% had recovered, 14% partially recovered, and 73% remained depressed. In the adult population, the Netherlands Mental Health Survey [10] showed that 76% of participants recovered within 12 months and nearly 20% had not recovered at 24 months. These variations between studies, which could be attributable to different definitions of recovery and population differences, make comparison between studies difficult. However, there are few longitudinal population-based reports of depression trajectories in older adults.

Concerning predictors over time of adult depression, previous research has focused primarily on the clinical features of the disease and revealed that some factors such as stressful life events combined with genetic vulnerability were strongly related to the occurrence of depression [11]. 

However, research is scarce about social predictors of depressive disorder, among community-dwelling older adults. For example, a prospective community-based study, using a large sample of elderly people, has found a strong association of physical health and disability with depression [12]. With respect to psychosocial variables, most research has focused on marital status and social support [13, 14] and showed that marital distress and low social support are risk factors for depression [15–17]. In a clinical sample of older depressed patients, low social participation was found to be associated with worse outcomes [16]. However, the influences of sociodemographic, health, and psychosocial factors on the development of depression in older adults were not examined together. Given the increase in the ageing population and the paucity of data characterizing patterns of depression in community-dwelling older adults, it is important to conduct longitudinal studies that can better characterize how depressive disorder changes in older individuals over time and identify the factors that may be associated with these changes.

Our objective was to describe changes in depressive disorder in community-dwelling older adults over a 12-month period and to identify the social factors on outcomes in three groups of depressed patients (continuously depressed, recovered, and incident depression) compared to non depressed elderly.

## 2. Methods

### 2.1. Study Population and Data Collection

This research is part of the longitudinal ESA Study (Enquête sur la Santé des Aînés) conducted in 2005–2008, in a large cohort of community-dwelling older adults aged 65 years and more (*n* = 2752) followed over a 12-month period. Data was collected through at-home, face-to-face interviews. The study population, data collection procedures, and ethics are described in previous publications [18–20]. Briefly, a probabilistic sample of French-speaking community-dwelling older adults (94% of the population of Quebec who are able to understand and speak French) was used. Respondents living in northern regions of Quebec were excluded on feasibility grounds.

The response rate for this study was 78.5% at time 1 and 79.1% of the respondents at time 1 participated in the follow-up interview 12 months later.

## 3. Measures 

### 3.1. Dependent Variable

Depressive disorder (including major and minor depression) according to criteria from the DSM-IV (Diagnostic and Statistical Manual of Mental Disorders, Fourth Edition) [21] was measured using a computer questionnaire, the ESA Diagnostic Questionnaire (ESA-Q) developed by the research team [20]. In this study, a 12-month recall period was used. For the purpose of this study, and as reported in previous research [18, 19], a dichotomous outcome was defined as 1 if the person fulfilled the criteria for major or minor depression and 0 otherwise. Subjects who displayed the essential features of depression and reported between 2 and 4 of the 9 associated symptoms of depression were classified as minor depression cases.

The study was in accordance with the guidelines laid down in the current version the Statement of tri-Council Policy, approved by the Canadian Institutes of Health Research.

Written consent was obtained at the beginning of the interview and the project was approved by the Ethics Committee of the Sherbrooke University Geriatric Institute.

For the purpose of the study, the cohort was divided into four groups: (1) subjects who never met the criteria for depressive disorder at baseline and followup (reference group); (2) continuously depressed cases, which included subjects who presented symptoms meeting DSM-IV criteria for depressive disorder at both baseline and followup; (3) recovered cases, which included subjects who met DSM-IV criteria for depressive disorder at baseline but did not report all symptoms required for depression 12 months later; and (4) incident (new) cases, which included subjects who did not meet DSM-IV criteria for depressive disorder at baseline and reported symptoms related to depressive disorder, in the followup.

### 3.2. Independent Variables

#### 3.2.1. Demographic Characteristics

The interview collected standard demographic information about respondents, including age, gender, marital status, education, and region of residence. Marital status was measured as follows: single (never with a partner), living with a partner (including married and cohabiting with a partner), separated, and widowed. Education was broken down as follows: elementary (1–7 years), secondary (8–12 years), and post-secondary/university. Region of residence was divided into three categories: (1) metropolitan (city of Montreal), (2) urban, and (3) rural [22]. 

*Health status *was measured by morbidity and self-rated mental health. Morbidity was assessed by the number of chronic health problems as defined in the International Classification of Diseases, version 10 (ICD-10) (International Classification of Diseases, 10th Revision), calculated based on a list of 18 common chronic health problems, developed and revised by the World Health Organization [23] (arthritis, cardiac disease, disorders of the digestive system, endocrine system, diabetes, diseases of the kidney, liver, and eyes). Self-rated mental health was evaluated with the question: “In general, compared to other people of your age, would you say your mental health is generally excellent, very good, good, fair or poor”?

*Psychosocial factors* were measured by four indicators: (1) number of daily hassles, (2) intensity of daily hassles, (3) social support, and (4) social integration.

Daily hassles were measured with the Daily Hassles Scale, which examines irritating, frustrating daily demands that characterize every day transactions with the environment. This scale originally had 117 items [24]. A French version consisted of 64 items [25] each representing a specific hassle. For the purpose of the ESA Study, this scale was reduced to 30 items. This version was validated with 196 French-speaking seniors with a Cronbach alpha of 0.90. The test-retest reliability coefficients were 0.79 and 0.60 for the frequency of hassles and reported intensity, respectively.

The level of intensity as measured by each item ranged from 0 (not at all) to 5 (extremely). The sum of the 30 items was then divided by the total number of items to obtain the final intensity score (0 to 5), yielding a continuous variable.

Social support was measured using three questions (yes/no) from the Quebec Health and Social Survey [26]: (1) availability of a confidant to talk to about various problems, (2) presence of someone who could provide instrumental help, and (3) presence of someone who could provide emotional support. This social support measure has good construct validity and good predictive validity [26].

Social integration was measured using three questions (yes/no) on participation in the community: (1) Do you regularly go to leisure, cultural, or social centers? (2) Do you regularly do volunteer work? (3) Do you regularly attend religious services (church, synagogue, mosque, or other centers of worship)? 

## 4. Statistical Analysis

All analyses were carried out using SAS software (Statistical Analysis System: http://www.sas.com/).

We used polytomous logistic regression to assess the relationship between possible determinants at baseline and depression in individuals who are continuously depressed, those who recovered, and incident cases, compared to individuals who did not meet the criteria for depressive disorder at baseline and the follow-up interview 12 months later (reference group). Confidence intervals of 95% were estimated for the odds ratios for depression related to each risk factor.

## 5. Results

**Figure 1:**
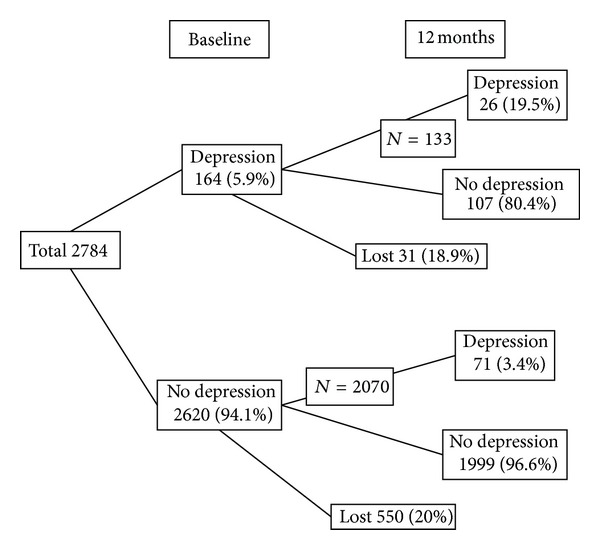
Depression patterns among the participants at baseline and the follow-up interview 12 months later (*n* = 2784).

Subjects lost to follow-up did not differ at baseline on depression, demographic, and other independent variables from those still participating 12 months later. Depression patterns among participants at baseline and the followup are shown in Figure 1.

Among the 164 (5.9%) participants who presented symptoms meeting DSM-IV criteria for depression at baseline, 26 (19.5%) of them met criteria for depression (continuously depressed cases) and 107 (80.4%) had recovered at the follow-up interview 12 months later. Among the 2620 subjects (94.1%) who did not present symptoms meeting DSM-IV criteria for depression at baseline, 71 (3.4%) presented symptoms meeting DSM-IV criteria for depression (incidentor recurrent cases) 12 months later. 

**Figure 2:**
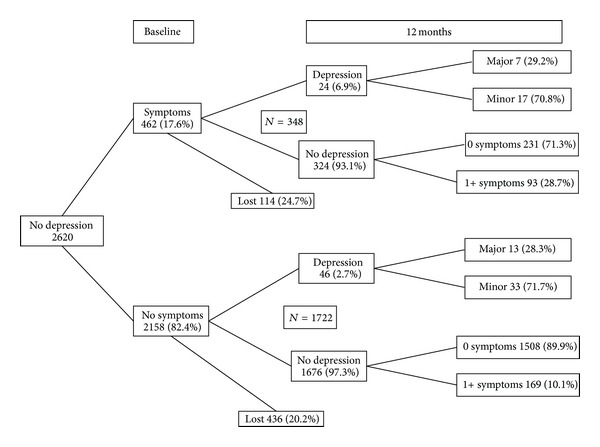
Depression symptom patterns among the participants who did not present symptoms meeting DSM-IV criteria for depression at baseline (*n* = 2620).

Depression symptom patterns among the 2620 respondents who did not present symptoms meeting DSM-IV criteria for depression at baseline are shown in Figure 2. 

Of the 462 (17.6%) presenting one or more subsyndromal depression symptoms at baseline, 24 (6.9%) presented symptoms meeting DSM-IV criteria for depression 12 months later compared to 46 (2.7%) of those who did not present any subsyndromal depression symptoms at baseline (Figure 2).

The results of polytomous logistic regression models are shown in Table 1. According to the multivariate model, several factors could increase the probability of all three possible depression states: number of daily hassles (continuously ill, OR = 1.09, 95% CI: 1.03 to 1.14; recovered, OR = 1.06, 95% CI: 1.03 to 1.09; recurrent, OR = 1.06, 95% CI: 1.02 to 1.09), stress intensity (continuously depressed, OR = 3.17, 95% CI: 1.97 to 5.10; recovered, OR = 2.02, 95% CI: 1.49 to 2.74; recurrent, OR = 1.72, 95% CI: 1.19 to 2.49), and fair/poor self-rated mental health (continuously ill, OR = 3.87 versus excellent/very good, 95% CI: 1.17 to 12.80; recovered, OR = 3.98, 95% CI: 1.78 to 8.93; recurrent, OR = 3.93, 95% CI: 1.49 to 10.38).

Continuity of depression was predicted by marital status (OR = 3.20 never with a partner versus living with a partner, 95% CI: 1.12 to 9.14; OR = 3.19 separated versus living with a partner, 95% CI: 1.27 to 7.99) and a high number of chronic diseases (OR = 1.17, 95% CI: 1.00 to 1.14). Recovery from and recurrence of depression (unstable condition) were both associated with two other factors: gender (female) (recovered, OR = 1.85, 95% CI: 1.10 to 3.10; incident, OR = 1.99, 95% CI: 1.08 to 3.68) and region of residence (living in rural region) (recovered, OR = 1.93, 95% CI: 1.18 to 3.16; incident, OR = 2.91, 95% CI: 1.58 to 5.36). 

Recovery from depression was predicted by marital status (OR = 2.05 separated versus living with a partner, 95% CI: 1.14 to 3.69) while incidence was predicted by not attending places of worship, the only social integration factor that significantly predicted depression status (OR = 1.99 attendance versus non attendance at places of worship, 95% CI: 1.13 to 3.51). 

No other variables were independently associated with any depression status after adjusting for covariates.

## 6. Discussion 

The objective of this study was to describe changes in depressive disorder and to examine the influence of sociodemographic, health, and psychosocial factors on later changes in depression leading to continuity of the depression recovery or incidence in community-dwelling older adults over a 12-month period. 

Our results revealed that among the 164 participants, who presented symptoms meeting DSM-IV criteria for depression at baseline, 19.5% were continuously depressed and 80.4% had recovered at the follow-up. This result is not consistent with those found in a Canadian study [9] which found that in elderly medical inpatients with major or minor depression, at the one-year followup, 13% had recovered, 14% partially recovered, and 73% remained depressed. This difference could be attributable to differences in the populations studied (community dwelling versus medical inpatients). Also, our results showed that the risk of depression over the 12-month period was higher among women, living in a rural region, with a large number of daily hassles, high level of stress intensity, high number of chronic diseases, and fair/poor self-rated mental health. 

At first glance, the results of polytomous logistic regression models showed great similarities between the risk factors of the three states. Number of daily hassles, stress intensity, and self-rated mental health were associated with all of them. This finding is consistent with those of another study in a sample of initially clinically depressed elderly patients [27]. These factors would not predict the type of changes in depression in older adults. They would simply indicate vulnerability to depression. Some longitudinal studies also found that the severity of life events [28] influences the outcomes of depression. Some other studies have identified the number of daily hassles as potential risk factors for persistence [29] while other studies did not find any association [30] or in the multivariate model this effect disappeared [31]. 

However, other factors could be used as indicators to predict changes in depression that already exists, or to identify vulnerability to potential depression.

The number of chronic diseases and marital status significantly predicted outcomes in the continuously ill subgroup, but not in the other subgroups. In agreement with our results, a quantitative meta-analysis [32] showed that elderly with chronic diseases and poor self-rated health had a higher risk of chronic or persistent depression. Also, in one study [27] results underline the importance of poor self-rated health as a predictor of longer time-to-remission. On the other hand, never having lived with a partner or being separated seems to be associated with lasting depression in the elderly population, as [13] observed earlier, but not with fluctuating moods. In the literature, the results are contradictory. Some other studies identified marital status as a potential risk factor for persistence of depression [29] but other research did not found any association [30]. However, our results suggest that when older depressive people are single or separated, have multiple chronic diseases, suffer from severe stress, report a high number of hassles, and consider themselves to be in poor mental health, they should receive special attention from health professionals. They are likely to be at greater risk of suffering from prolonged depression, which may become chronic. 

Finally, the results of polytomous logistic regression models showed some other factors, gender and region of residence more typically independently associated with mood instability, that is, recovering from (in remission) or relapsing into (recurrent) depression. Being a woman, living in a rural area, having numerous daily hassles, intense stress, and poor self-rated mental health significantly predicted mood swings. The majority of studies found a higher prevalence of depression in women [33]. Our results, however, add a nuance whereby older women do not seem to be at greater risk of persistent depression but do seem to suffer more from fluctuating emotional states, recovering from (remission) or relapsing into depression, at least among those living in the community [7]. Also, in agreement with a majority of studies [34] our results do not indicate an increased risk of persistent depression by region of residence. This factor appears to influence only emotional variability, with people in rural areas being at greater risk of having their mood improved or deteriorated, than people in a Metropolitan area. 

Two other factors also influence the stability of depression, but differently in different states. For the continuously ill, being separated increases the likelihood of a positive change towards recovery compared to being married, but to a lesser extent. On the other hand, non-attendance at places of worship seems to predict the recurrence of depression. This was the only independent significant social relationships factor in our study. This result is not consistent with those found in other studies [35] and revealed that lower instrumental and perceived social support was associated with longer time to remission of geriatric depression in bivariate analyses, but this association between confident and instrumental support disappears when taking into account proximal variables such as hassles and stress. When their clients suffer from mood swings, health professionals should pay particular attention to both the difficulties and the opportunities in their region of residence, their marital status and their sex (woman or not) to sustain a remission, and also the lack of support associated with not attending places of worship in the case of a possible recurrence. 

## 7. Strengths and Limitations

Longitudinal studies on psychosocial factors and depression are limited. This study contributes knowledge on the natural course of depression over one year in a large population-based cohort of community-dwelling older adults in Quebec by examining the frequency of persistence, recovery, and incidence and the influence of sociodemographic, medical, and psychosocial factors together on the development of depression in the elderly.

However, our study has some limitations. The follow-up (one year) is somewhat short to assess the longitudinal association between the independent variables and outcomes, although the time between depressive episodes is shorter in the elderly than in adults [36, 37]. Also, the number of subjects in the continuously ill subgroup is limited. In addition, we lack information on disability, an important risk factor for depression incidence [38, 39], and social support variables were measured as dichotomous instead of in a continuous scale which hinders the study of their association with depression states. 

## 8. Conclusion

Depression in old age is dynamic. Results support the hypothesis about psychosocial factors as predictors over time of depression, in old persons. High level of stress intensity and high number of chronic diseases, associated with chronicity and recovery, should be taken into account when diagnosing and treating community living older people.

These results highlighted a need for future longitudinal studies to confirm the predictive influence of these variables and to evaluate their effect on diagnosis and treatment of depression.

## Figures and Tables

**Table 1:** Multivariate polytomous regression models predicting depression' outcomes at time two.

	Persistent		Remission		Incident	
	OR	95% CI	OR	95% CI	OR	95% CI
Gender						
Male	1.00	—	1.00	—	1.00	—
Female	0.58	0.28–1.21	1.85	1.10–3.10	1.99	1.08–3.68
Age						
65–69 years	1.00	—	1.00	—	1.00	—
70–74 years	1.07	0.47–2.42	1.07	0.62–1.85	0.84	0.44–1.62
75–79 years	0.57	0.20–1.57	0.84	0.45–1.56	0.60	0.27–1.35
80+ years	0.40	0.12–1.34	0.78	0.39–1.57	1.09	0.50–2.33
Education						
Postsecondary/university	1.00	—	1.00	—	1.00	—
Elementary	0.95	0.35–2.55	0.61	0.32–1.17	0.95	0.44–2.05
Secondary	1.37	0.60–3.16	1.15	0.69–1.92	1.36	0.72–2.59
Marital status						
Living with a partner	1.00	—	1.00	—	1.00	—
Single	3.20	1.12–9.14	0.48	0.15–1.50	0.72	0.23–2.24
Separated	3.19	1.27–7.99	2.05	1.14–3.69	0.40	0.14–1.17
Widowed	1.17	0.48–2.87	0.83	0.49–1.42	0.61	0.32–1.16
Region of residence						
Metropolitan	1.00	—	1.00	—	1.00	—
Urban	1.15	0.40–3.28	1.46	0.74–2.88	1.63	0.66–3.99
Rural	1.26	0.59–2.71	1.93	1.18–3.16	2.91	1.58–5.36
Presence of confident						
Yes	1.00	—	1.00	—	1.00	—
No	0.67	0.20–2.17	1.31	0.68–2.53	1.99	0.97–4.09
Instrumental support						
Yes	1.00	—	1.00	—	1.00	—
No	2.40	0.61–9.54	1.62	0.64–4.09	2.40	0.80–7.14
Emotional support						
Yes	1.00	—	1.00	—	1.00	—
No	1.49	0.35–6.39	1.25	0.48–3.23	0.31	0.07–1.42
Participation in community centers						
Yes	1.00	—	1.00	—	1.00	—
No	1.00	0.49–2.04	0.71	0.46–1.11	1.50	0.84–2.69
Volunteering						
Yes	1.00	—	1.00	—	1.00	—
No	1.32	0.60–2.91	1.19	0.73–1.93	0.88	0.48–1.61
Attendance at places of worship						
Yes	1.00	—	1.00	—	1.00	—
No	0.59	0.29–1.24	0.72	0.45–1.15	1.99	1.13–3.51
Number of daily hassles	1.09	1.03–1.14	1.06	1.03–1.09	1.06	1.02–1.09
Stress intensity	3.17	1.97–5.10	2.02	1.49–2.74	1.72	1.19–2.49
Number of chronic disease	1.17	1.00–1.36	1.07	0.97–1.19	1.06	0.94–1.21
Perception of mental health						
Excellent/very good	1.00	—	1.00	—	1.00	—
Good	3.68	1.74–7.78	1.82	1.08–3.07	1.91	1.03–3.55
Fair/poor	3.87	1.17–12.80	3.98	1.78–8.93	3.93	1.49–10.38
